# Classification of the human THAP protein family identifies an evolutionarily conserved coiled coil region

**DOI:** 10.1186/s12900-019-0102-2

**Published:** 2019-03-05

**Authors:** Hiral M. Sanghavi, Sairam S. Mallajosyala, Sharmistha Majumdar

**Affiliations:** 1 Discipline of Biological Engineering, Indian Institute of Technology Gandhinagar, Gandhinagar, India; 2 Discipline of Chemistry, Indian Institute of Technology Gandhinagar, Gandhinagar, India

**Keywords:** THAP proteins, Classification, Leucine zipper, Oligomerization

## Abstract

**Background:**

The THAP (Thanatos Associated Proteins) protein family in humans is implicated in various important cellular processes like epigenetic regulation, maintenance of pluripotency, transposition and disorders like cancers and hemophilia. The human THAP protein family which consists of twelve members of different lengths has a well characterized amino terminal, zinc-coordinating, DNA-binding domain called the THAP domain. However, the carboxy terminus of most THAP proteins is yet to be structurally characterized. A coiled coil region is known to help in protein oligomerization in THAP1 and THAP11. It is not known if other human THAP proteins oligomerize. We have used bioinformatic tools to explore the possibility of dimerization of THAP proteins via a coiled coil region.

**Results:**

Classification of human THAP protein into three size based groups led to the identification of an evolutionarily conserved alpha helical region, downstream of the amino terminal THAP domain. Secondary structure predictions, alpha helical wheel plots and protein models demonstrated the strong possibility of coiled coil formation in this conserved, leucine rich region of all THAP proteins except THAP10.

**Conclusions:**

The identification of a predicted oligomerization region in the human THAP protein family opens new directions to investigate the members of this protein family.

**Electronic supplementary material:**

The online version of this article (10.1186/s12900-019-0102-2) contains supplementary material, which is available to authorized users.

## Background

The THAP (Thanatos Associated Proteins) protein family is characterized by a conserved amino-terminal zinc-coordinating DNA-binding domain [1]. The THAP protein family in humans consists of twelve members that vary in size from 200 to 900 amino acid residues. THAP7 and THAP11 have been characterized as transcription factors [2, 3]. Other THAP proteins have been implicated in diverse cellular responses; THAP0 is a member of the apoptotic cascade induced by IFN-γ [4], THAP1, with RRM1, regulates cell proliferation [5], THAP5 is a cell cycle inhibitor [6], and THAP9 is an active transposase in humans [7]. The THAP11 homologue in mice is essential for pluripotency in mice [3]. THAP proteins have also been linked to various diseases: THAP1 is implicated in torsional dystonia and hemophilia [8], THAP5 is implicated in several heart diseases [9, 10] and THAP2, 10 and 11 are implicated in various cancers [9].

Coiled coils are structural features in proteins that mediate protein oligomerization and are characterized by amphipathic alpha helices of each monomer twisting around each other. They are often made of a repeated pattern of seven amino acids called a “heptad repeat” which fold into amphipathic alpha helices [11]. If the amino acids in each heptad are labelled from *a* to *g*, non-polar/hydrophobic residues usually occur at every *a* and *d* position and charged amino acids at every *e* and *g* position (Additional file 1: Figure S1a). Leucine zippers are coiled coil regions which predominantly have leucine in the *d* position of the heptad repeat. The side chains of the hydrophobic residues at *a* and *d* on each monomer strand undergo ‘knobs-into-holes’ packing [12] by interlocking with a similar pattern on another monomer strand to form a hydrophobic core. Helical regions of proteins can be visually represented by a helical wheel plot (Additional file 1: Figure S1b) wherein the amino acid sequence of the protein is plotted in a rotating manner around a central axis [13].

Coiled coils enable oligomerization of various proteins like signal transducers, transcription factors, actin and many more [14–16]. Protein oligomerization is observed in most cellular processes like formation of cytoskeleton, cell signal transduction, regulation of gene expression, transposition [16, 17]. Proteins can undergo homo-oligomerization (binding itself) or hetero-oligomerization (binding other protein interaction partners).

Formation of homo-oligomers is commonly seen in transcription factors [15]. Biochemical evidence suggests that THAP proteins may undergo homo dimerization. THAP0, also known as PRKRIR and Death Associated Protein 4, forms a homodimer using amino acid residues 1–488 [4, 18]. However, there are no structural studies which report the formation of coiled coils in THAP0. Mutation studies on THAP1 demonstrate the formation of a coiled coil region (residues 139–190) which is indispensable for THAP1 homo dimerization [8]. The recently reported carboxy terminal coiled coil region of human THAP11 (residues 254–306, PDB id: 5AJS) has been shown to form parallel homo dimers [19].

Hetero-dimerization of proteins also has important functional consequences as seen in cell shape determining proteins of *H. pylori* [20] and SNARE (soluble NSF Attachment Receptors) in yeast and mammals [21]. Some human THAP proteins are reported to form heterodimers with HCF-1 [22]. THAP0 binds MST1 [4], THAP3 shares sequence similarity and protein interaction partners with THAP1 [22]. THAP7 binds to hypo-acetylated histone H4 tails via its carboxy terminal 77 amino acid residues (residues 232–309). The THAP domain and the Histone interacting domain (HID, a predicted coiled coil region of THAP7) play key roles in binding TAF- 1β and transcriptional repression [2].

The carboxy terminal portions of most THAP proteins, except for THAP1 and THAP11, are yet to be structurally and functionally characterized [8, 19]. In this study, we explore the possibility of oligomerization of THAP proteins by predicting a coiled coil region in a ~ 40 amino acid leucine rich region that is located downstream of the highly conserved DNA-binding THAP domain.

## Methods

### Secondary structure prediction

Secondary structures of THAP proteins were predicted using JPRED [23], PSIPRED [24] and Phyre2 [25]. Briefly, JPRED constructs a Multiple Sequence Alignment using PSI-BLAST [26] for individual input sequences and uses it to predict local secondary structure using Jnet [27]. PSIPRED generates a sequence similarity search using PSI-BLAST [26] and then predicts the secondary structure using artificial neural network machine learning approach, followed by filtering the predicted secondary structures using two separate neural networks followed by actual structure prediction. Phyre2 scans the sequence using PSI-BLAST [26], followed by secondary structure prediction using the neural network secondary structure prediction.

### Multiple sequence alignment of the THAP proteins

Amino acid sequences of the twelve THAP proteins were downloaded from NCBI (NP_004696.2, CAG33537.1, AAH08358.1, AAH92427.1, AAH69235.1, Q7Z6K1.2, AAH22989.1, NP_001008695.1, Q8NA92.1, NP_078948.3, NP_064532, NP_065190.2 and aligned using Clustal Omega [28]. Clustal Omega allows identification of conserved and similar amino acids among the input sequences based on HMM models.

### Sequence conservation score analysis of THAP proteins

Sequence conservation score for each position of the predicted coiled coil region of THAP proteins were generated using “Protein Residue conservation Prediction” [29]. Briefly, the conservation of an amino acid at a position when aligned with similar protein sequences indicates significant evolutionary pressure at that position. This is quantified using Jensen-Shannon divergence (JSD) and is combined with a window based extension method to take into account the conservation of sequentially adjacent residues.

### Generation of protein models

Protein models were generated by I TASSER [30], RaptorX [31] and LOMETS [32]. Briefly, the amino acid sequences of the THAP proteins were submitted to the I TASSER server, RaptorX server and LOMETS server with no specified template. Suitable templates depending on sequence similarity searches were identified from the PDB database. Monte Carlo simulations were used to assemble the full- length conformations of identified templates and models were generated for the sequence of interest. Lastly, all the conformations were confirmed and cluster centroids were identified which were then used to build the final models after refinement of cluster centroids.

### Helical wheel plot analysis

Helical wheel plots of the selected residues of THAP proteins were generated using ‘DRAW COIL’ [33]. Briefly, a helical wheel plot visualizes the arrangement of amino acids in a helical wheel pattern i.e. if a protein has strong probability of forming a coiled coil, hydrophobic amino acids cluster to one side of the helical wheel plot and hydrophilic amino acids cluster on the opposite side (amphipathic pattern), which is common for proteins forming leucine zippers. In addition to predicting amphipathic pattern, DRAW COIL also predicts probable hydrophobic and electrostatic interactions between the amino acid residues of a homodimer.

### Higher order oligomeric structure prediction

The possibility of forming higher order oligomeric structures by the predicted alpha helical regions of THAP proteins (except THAP10) and their respective interacting partners indicated by STRING database [34] was predicted by Multicoil [35] and LOGICOIL [36]. Briefly, Multicoil predicts a probable dimer or a trimer by calculating the pairwise frequency values of a given amino acid residue pair in the input peptide or protein sequence and comparing it to the pairwise frequency values available from the established coiled coil data. LOGICOIL use Bayesian variable selection along with multinomial probit regression method to predict the formation of a higher order oligomeric structure like parallel or anti-parallel dimer, trimer or a tetramer of a given protein/peptide sequence.

## Results

### Prediction of leucine-rich alpha helical regions in THAP proteins by secondary structure analysis and multiple sequence alignment

The probability of secondary structure formation of human THAP proteins was predicted by JPRED, PSIPRED and Phyre2. Analysis of results from all three secondary structure prediction tools identifies a region spanning around 40 amino acid residues downstream of the THAP domain in each THAP protein (except THAP10), that has a high probability of forming alpha helices, as shown in Table 1.

**Table 1:** Alpha helical regions predicted in human THAP proteins using JPRED, PSIPRED and Phyre2

THAP protein	Residue range	Start residue	End residue
THAP0	31	150	180
THAP1	46	143	188
THAP2	44	135	178
THAP3	36	189	224
THAP4	37	362	398
THAP5	42	331	372
THAP6	46	148	193
THAP7	45	237	281
THAP8	30	180	209
THAP9	38	145	182
THAP10	–	–	–
THAP11	57	254	310

Average length of predicted alpha helical region: 41.091

The predicted alpha helical regions correspond to residues 150–180 in THAP0, which is a part of the biochemically studied larger region spanning residues 1–488 [4, 18], residues 143–188 in THAP1 which overlaps with the experimentally verified coiled coil region (residues 139–190) [8], residues 135–178 in THAP2, residues 189–224 in THAP3, residues 362–398 in THAP4, residues 331–372 in THAP5, residues 148–193 in THAP6, residues 237–281 in THAP7, which overlaps with the predicted HID [2] in THAP7, residues 180–209 in THAP8, residues 145–182 in THAP9 and residues 254–310 in THAP11, which is a part of the previously reported X ray crystal structure of the coiled coil region (residues 247–314) of THAP11 [19].

The predicted alpha helical region in THAP0, THAP3 THAP4, THAP5, THAP6, THAP7, THAP8, THAP9 and THAP11 are rich in leucine (Additional file 1: Table S1) and hydrophobic residues as seen in the characteristic heptad repeats of coiled coils. However, THAP11 is an exception to the general rule since it does not have a leucine at every *d* position but still forms a coiled coil region (residues 254–306) [19]. The corresponding regions in THAP1 and THAP2 are leucine poor (Additional file 1: Table S1).

### The human THAP protein family was divided into three groups based on conservation in predicted alpha helical regions

**Fig. 1:**
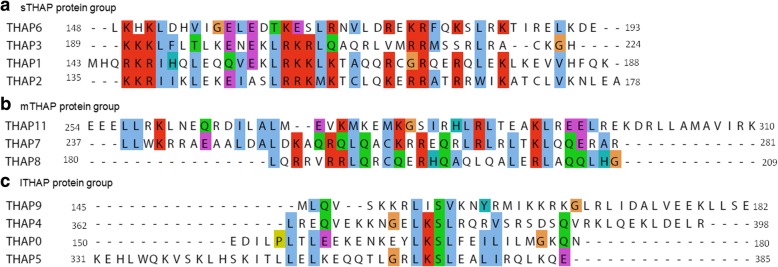
Multiple sequence alignment of leucine-rich alpha helical regions of human THAP proteins. The leucine rich alpha helical regions in human THAP proteins align with each other within THAP protein groups. **a** Helical region corresponds to residues143–188 (THAP1), 135–178 (THAP2), 189–224 (THAP3) and 148–193 (THAP6) (**b**) Helical region corresponds to residues 237–281 (THAP7), 180–209 (THAP8) and 254–310 (THAP11) (**c**) Helical region corresponds to residues 150–180 (THAP0), 362–398 (THAP4), 331–372 (THAP5) and 145–182 (THAP9). Boundaries of each protein are labelled. The Multiple Sequence Alignments were generated using CLUSTAL OMEGA and visualized using Jalview [47]. The color coding in Jalview is as follows; If each column has more than 60% hydrophobic amino acid residues, it is represented by blue, red represents more than 60% positively charged amino acid residues combined or more than 80% of either of the positively charged amino acid residues, more than 60% of negatively charged amino acid residues are represented by magenta, around 50 to 60% of polar amino acid residues are represented by green, Cysteine amino acid is pink colored, Glycine amino acid is orange colored, Proline amino acid is represented by yellow, more than 60% of aromatic amino acid residues are colored cyan and unconserved amino acid residues are represented as white

Multiple sequence alignment of the predicted alpha helical regions of all the THAP proteins (except THAP10) did not show any conservation of specific amino acid residues as shown in Additional file 1: Figure S2. However, multiple sequence alignment of THAP1, THAP2, THAP3 and THAP6 revealed strong conservation of basic amino acid residues (Fig. 1a). Similarly, multiple sequence alignment of THAP7, THAP8 and THAP11 proteins identified two conserved Leu residues and basic amino acid residues (Fig. 1b). Leu and Ser were found to be conserved upon multiple sequence alignment of THAP0, THAP4, THAP5 and THAP9 (Fig. 1c).

**Fig. 2:**
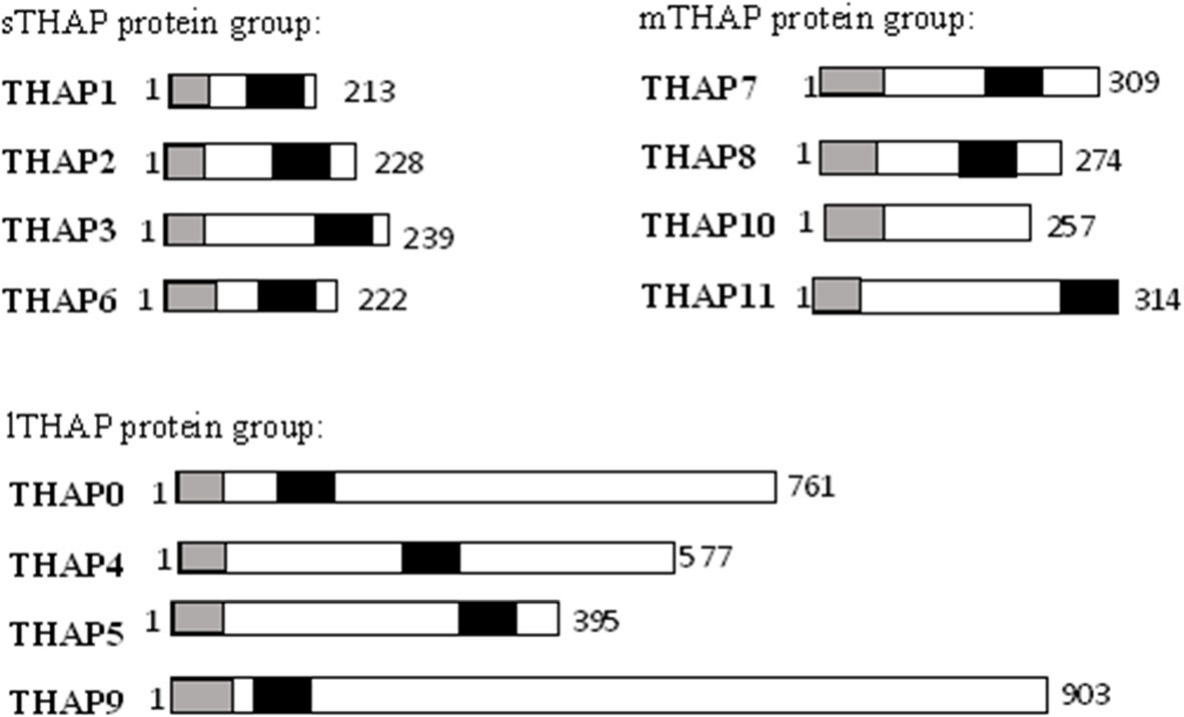
Classification of the human THAP protein family. The human THAP protein family is classified into three groups; Short THAP proteins include THAP1, THAP2, THAP 3 and THAP 6, Medium sized THAP proteins include THAP7, THAP 8, THAP 10 and THAP 11 and Long THAP proteins include THAP0, THAP 4, THAP 5 and THAP 9. Boundaries of each protein are labelled, grey box represents the THAP domain and black box represents the evolutionarily conserved coiled coil region, which has been predicted in this study

To obtain a more conserved picture over the entire THAP family, we classified the THAP proteins into three groups, based on the conservation found among the predicted alpha helical regions (1) Short THAP proteins (sTHAP): which include THAP1, THAP2, THAP3 and THAP6. (2) Medium sized THAP proteins (mTHAP): which include THAP7, THAP8, THAP10 and THAP11. (3) Long THAP proteins (lTHAP): which include THAP0, THAP4, THAP5 and THAP9 as shown in Fig. 2. Interestingly, the above classification also followed a length wise classification of all the THAP proteins i.e. Short THAP proteins (sTHAP): which have a length of less than 250 amino acid residues a (2) Medium sized THAP proteins (mTHAP): which have a length of less than 350 amino acid residues (3) Long THAP proteins (lTHAP): which have a length of more than 350 amino acid residues as shown in Fig. 2.

It is to be noted that the sequence conservation within the predicted alpha helical regions is only observed on classification of the THAP protein family. Multiple sequence alignment of all twelve full length THAP proteins did not yield any significant similarity downstream of the AVPTIF motif, which is speculated to be the carboxy- terminal boundary of the THAP domain, as shown in Additional file 1: Figure S3a. Moreover, inter-group multiple sequence alignment of the predicted alpha helical regions of sTHAP with mTHAP protein groups (Additional file 1: Figure S3b, sTHAP with lTHAP protein groups (Additional file 1: Figure S3c, and mTHAP with lTHAP protein groups (Additional file 1: Figure S3d) did not show any conservation of specific amino acid residues.

**Fig. 3:**
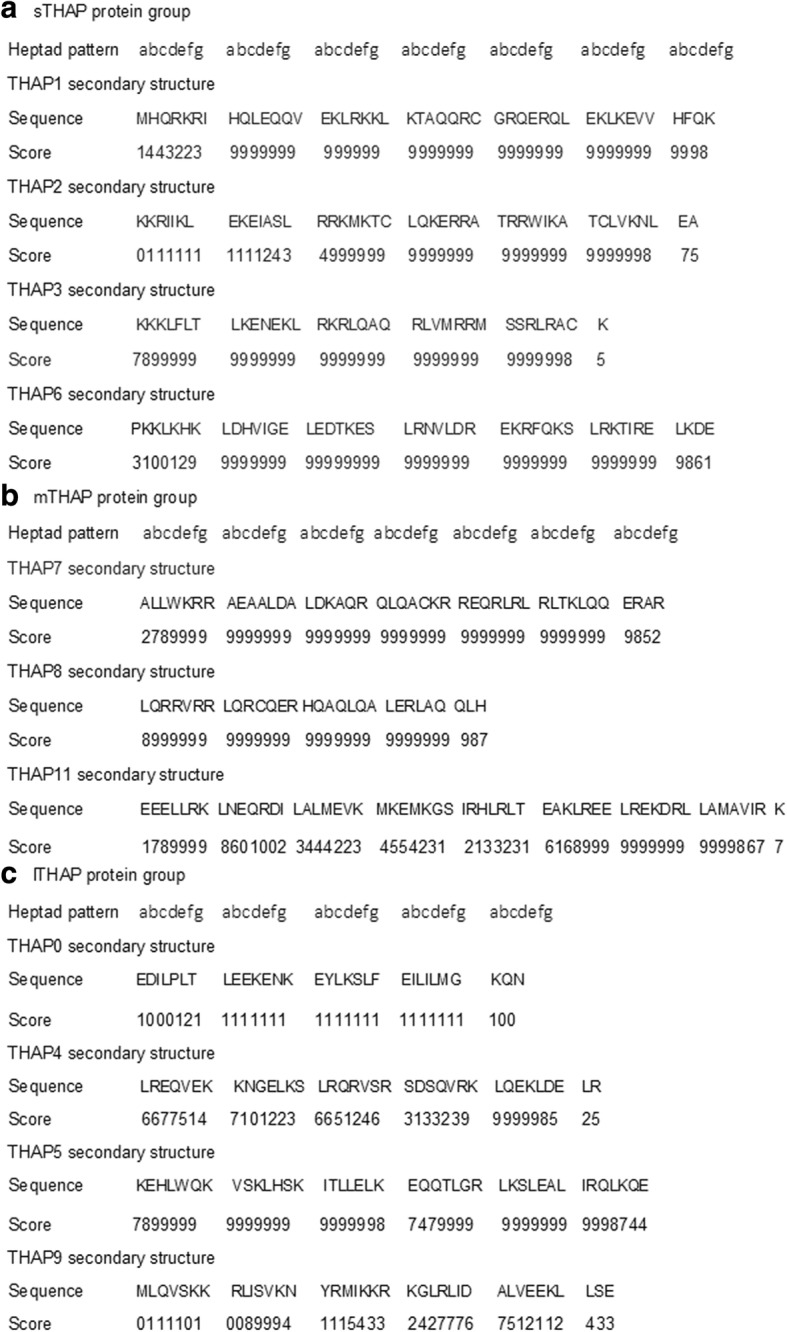
Prediction of secondary structure of THAP proteins. Predicted alpha helical regions are (**a**) sTHAP protein group: residues 143–188 (THAP1), 135–178 (THAP2), 189–224 (THAP3) and 148–193 (THAP6) (**b**) mTHAP protein group: residues 237–281 (THAP7), 180–209 (THAP8) and 254–310 (THAP11) (**c**) lTHAP protein group: residues 150–180 (THAP0), 362–398 (THAP4), 331–372 (THAP5) and 145–182 (THAP9). The secondary structure predicted by JPRED assigns a score from 0 to 9 for a predicted helix forming region, 0 being the lowest and 9 being the highest probability of forming a helix. The Heptad pattern is as described in Additional file 1: Figure S1a

**Fig. 4:**
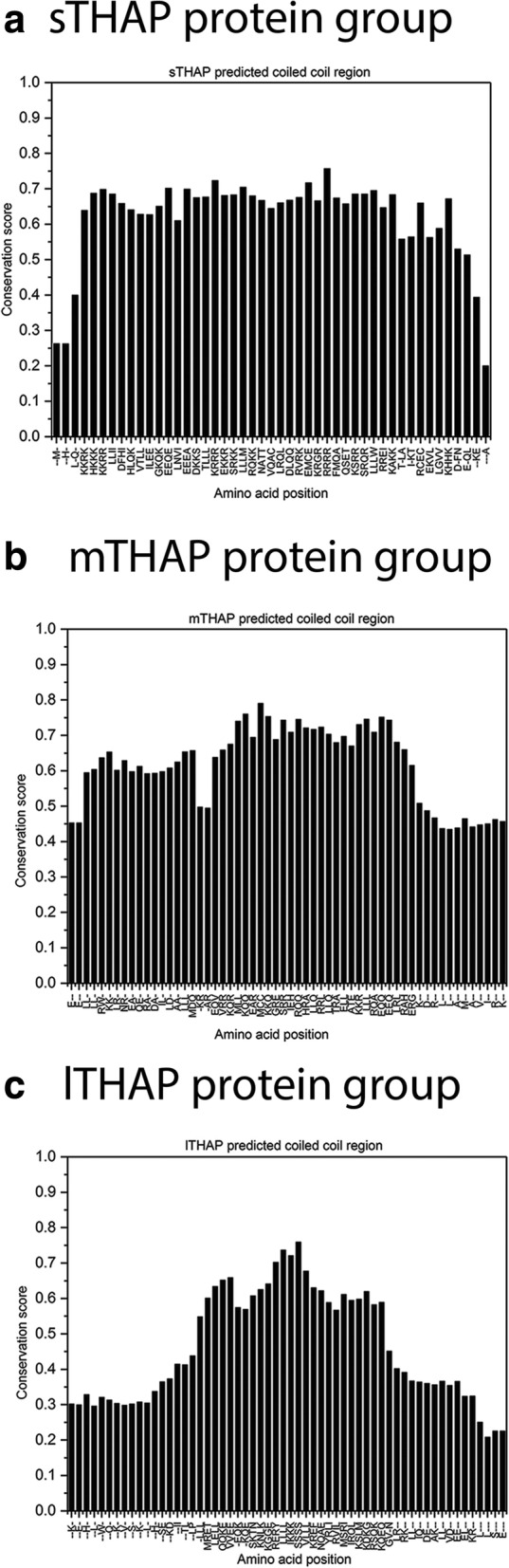
Group-wise protein residue conservation of predicted alpha helical region. **a** sTHAP protein group: THAP1, THAP2, THAP3 and THAP6 (**b**) mTHAP protein group: THAP7, THAP8, and THAP11 (**c**) lTHAP protein: THAP0, THAP5, and THAP9. The graph shows the conservation probability of each amino acid residue

The conserved amino acid sequences of the predicted alpha helical regions of the sTHAP protein group (Fig. 3a), mTHAP protein group (Fig. 3b) and lTHAP protein group (Fig. 3c) when aligned independent of the flanking regions of the proteins, demonstrated the heptad pattern that characterise coiled coil regions [21]. The high sequence conservation score (above 0.4) of the entire predicted alpha helical region of sTHAP group proteins (Fig. 4a) and mTHAP group proteins (Fig. 4b) indicate strong evolutionary conservation pressure in this region. The sequence conservation score is high (above 0.4) in the center of the predicted alpha helical region of lTHAP group proteins. This indicates a strong evolutionary conservation pressure within the predicted alpha helical region of lTHAP group proteins (Fig. 4c).

### Prediction of alpha helical regions in THAP protein models generated by threading-based models

**Fig. 5:**
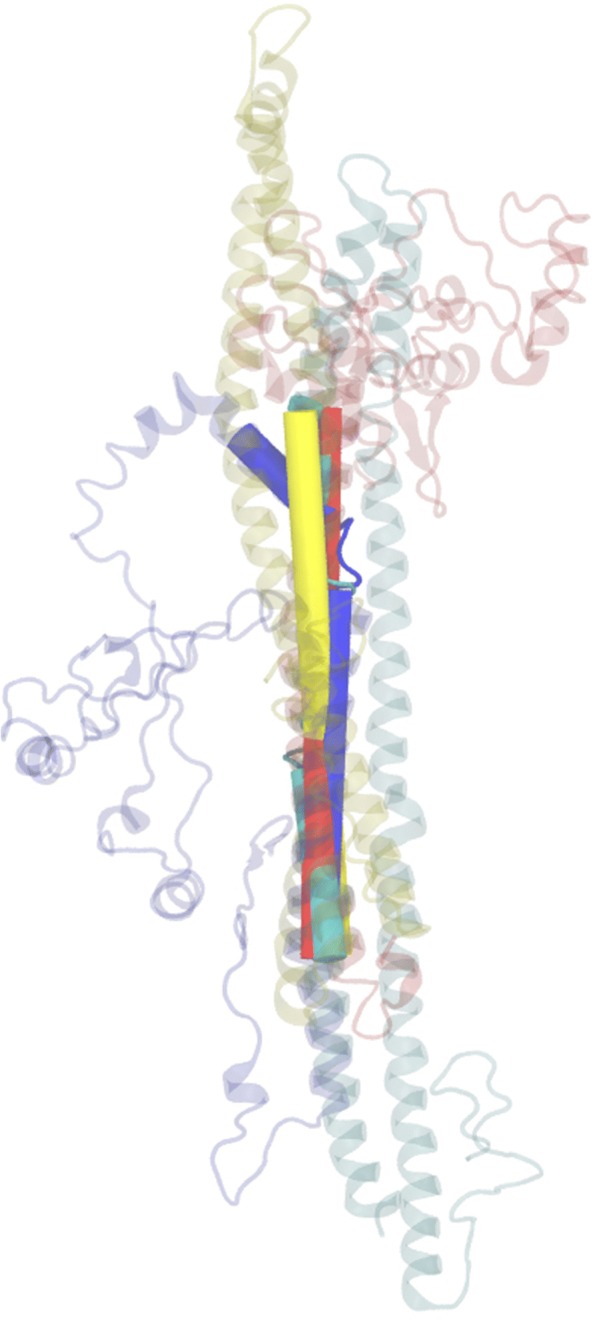
Superposition of the predicted alpha helical region of sTHAP protein group. The predicted alpha helical regions of sTHAP group proteins; THAP1 (143–188), THAP2 (135–180), THAP3 (180–225) and THAP6 (148–193) were represented as cartoon within the rest of each protein represented as ribbon and superposed using VMD. Green represents THAP1 protein, blue represent THAP2 protein, red represents THAP3 protein and yellow represents THAP6 protein

The structures of the human THAP proteins (except THAP10) were predicted using I TASSER, RaptorX and LOMETS (Additional file 1: Figure S4 Panel A. However, the threading-based models generated by LOMETS and I TASSER are more compact than the ones predicted by RaptorX and thus we use the I TASSER predicted protein models to visualize the predicted alpha helical secondary structures (Additional file 1: Figure S4 Panel B). It was interesting to note that the alpha helical regions predicted by I TASSER in each THAP protein (Additional file 1: Figure S4, Panel B), overlapped with the alpha helical region predicted by secondary structure prediction tools (Table 1, Fig. 3) and the region of amino acid similarity predicted by multiple sequence alignment (Fig. 1). The structural superposition of the predicted alpha helical regions of the sTHAP (Fig. 5) and mTHAP protein (Additional file 1: Figure S5) groups indicate structural similarity. The structure predictions for longer proteins are rather poor and less reliable. Thus, we do not attempt to superpose the predicted alpha helical regions of the lTHAP group.

This suggests that the potential to form alpha helices in these ~ 40 amino acid spanning regions may be independent of the folding of the flanking regions of the THAP proteins. Furthermore, the predicted helical region in THAP11 (residues 254–310; Table 1, Additional file 1: Figure S4, Panel B) is a part of the previously reported X ray crystal structure of the coiled coil region (residues 247–314) of THAP11 [19] (Additional file 1: Figure S6).

### Predicted alpha helical regions of THAP proteins arranged in an amphipathic pattern in helical wheel plots

Coiled coil regions mediate protein oligomerization wherein amphipathic alpha helices of each monomer twist around each other. DRAW COIL was used to investigate the presence of amphipathic pattern (arrangement of hydrophobic amino acid residues on one side of the alpha helical region and polar amino acid residues on the other side of the helix, when viewing the helix from the top) of amino acid residues and electrostatic interactions between charged amino acid residues within the predicted alpha helical regions in all THAP proteins, except for THAP10.

**Fig. 6:**
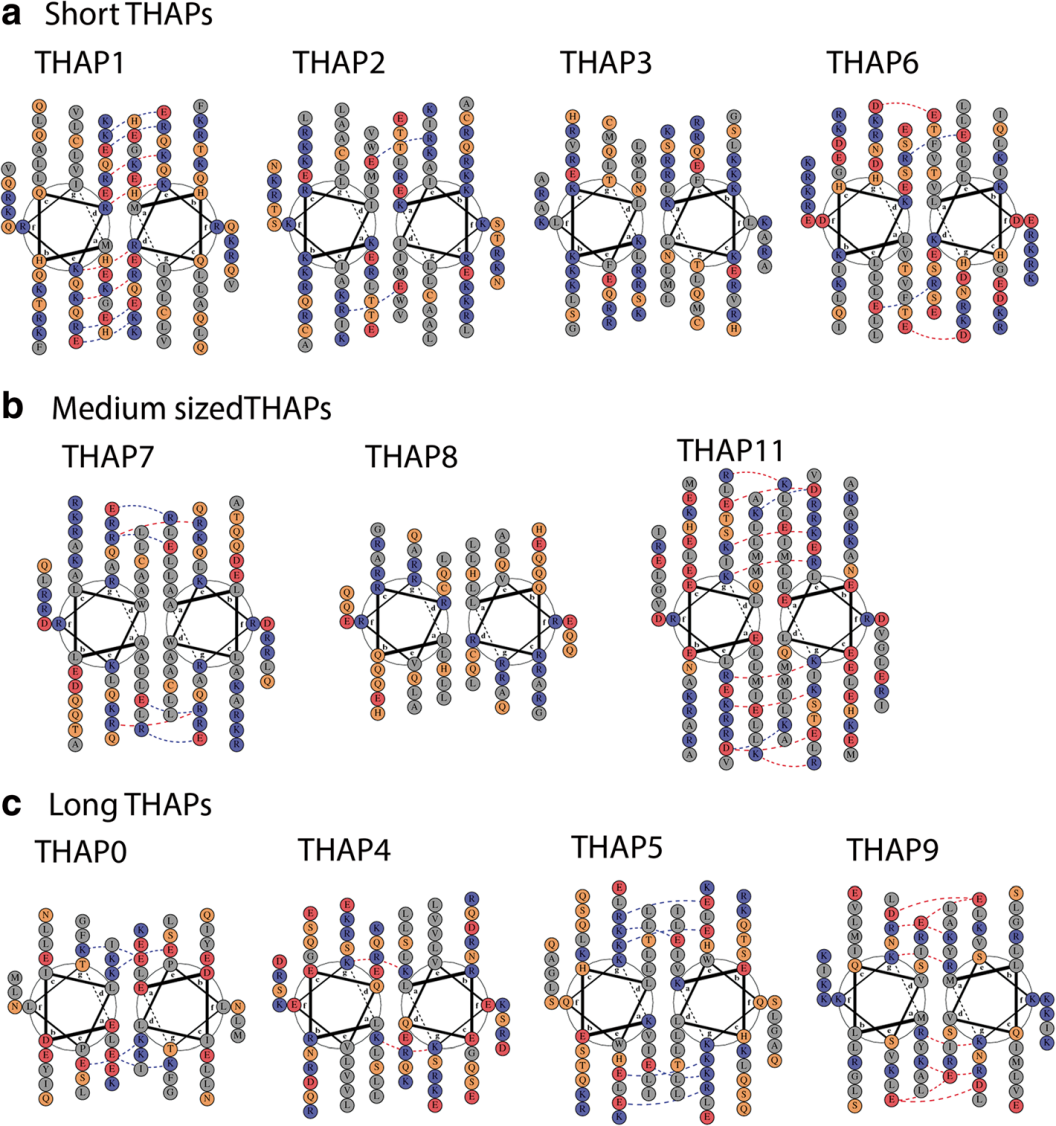
Helical wheel plot representation and electrostatic interactions predicted by DRAW COIL (**a**) sTHAP protein group: THAP1, THAP2, THAP3 and THAP6 (**b**) mTHAP protein group: THAP7, THAP8, and THAP11 (**c**) lTHAP protein: THAP0, THAP5, and THAP9. Helical wheel plots show the arrangement of amino acid residues in a heptad pattern. Non-polar residues are colored grey, polar residues are colored yellow, acidic residues are colored red and basic residues are colored blue. The red dotted lines represent electrostatic repulsive interactions and the blue dotted lines represent electrostatic attractive interactions

In the sTHAP protein group, the predicted alpha helical regions of THAP1 show a very strong amphipathic arrangement by clustering of non-polar amino acid residues (depicted by grey) on the top of the helical wheel and clustering of polar, acidic and basic amino acids residues (depicted by yellow, red and blue respectively) on the bottom of the helical wheel as seen in Fig. 6a. However, the predicted alpha helical regions of THAP2 and THAP3 of the sTHAP group (Fig. 6a), THAP8 of the mTHAP protein group (Fig. 6b) and THAP0 and THAP9 of the lTHAP group (Fig. 6c) show moderate amphipathic arrangement, whereas the predicted alpha helical regions of THAP4, THAP 5, THAP6, THAP7 and THAP11 (Fig. 6a, b,c) show very little amphipathic arrangement.

Within the predicted alpha helical region of the sTHAP protein group, interactions were predicted between Glu174 and Arg175 and Lys181 and Glu182 in THAP1, Glu158 and Arg159 in THAP2 and between Arg161 and Lys166 and Arg175 and Glu176 in THAP6, as shown in Fig. 6a. Similarly, within the mTHAP proteins group, interactions were predicted between Arg 264 and Glu 265 and Glu 279 and Lys 280 in THAP7 and Lys299 and Asp300 in THAP11 as shown in Fig. 6b. The interaction between Lys299K and Glu300 is reported to be very important for THAP11 dimerization as reported earlier [19]. In the lTHAP protein group, interactions were predicted between Lys 160 and Glu 161 and Lys163 and Glu164 in THAP0, between Lys344 and Glu349, Lys351 and Glu 352, Arg358 and Glu363 in THAP5 as shown in Fig. 6c. No electrostatic attractive interactions were predicted in the predicted alpha helical regions of THAP 3,4, 8, 9 (Fig. 6a, b, c).

### Predicted alpha helical regions are highly likely to form higher order oligomeric structures

The predicted alpha helical regions of all the THAP proteins except for THAP10 have a high probability to form higher order oligomeric structures as predicted by LOGICOIL and Multicoil. Interestingly, most of the interacting partners of THAP proteins (except THAP10) as predicted by STRING database also have a high probability of forming higher order oligomeric structures (Additional file 1: Table S2). This indicates that THAP proteins could form both homo- as well as hetero-oligomers.

## Discussion

We classify the human THAP protein family into three size based groups. Classification of proteins into families is used as a starting tool to get better insights into protein structure, function and evolutionary significance. CATH (class, architecture, topology, homologous superfamily) [37] and SCOP (Structural Classification of Proteins) [38] databases classify protein families based on fold domain recognition approach whereas Pfam [39] is a database based on amino acid sequence based classification. The fold domain approach gives a global view of protein structure while the sequence based classification gives insights into the evolutionary relationship (convergent or divergent) amongst proteins of the family. Based on inter- and intra-group multiple sequence alignments, we suggest that the three groups of human THAP protein family have become evolutionary divergent.

We report putative coiled-coil forming regions in all the human THAP proteins except THAP10. Since the discovery of the human THAP protein family [1], experimental studies describe cellular functions of several THAP proteins [1–5], structures of their DNA binding domains (THAP1) [40] and coiled coil regions (THAP11) [19].

Although the members of the human THAP protein family differ in their overall structures and cellular functions, they appear to have evolutionarily conserved domains like the well characterized amino-terminal DNA-binding THAP domain. This is the first extensive computational study conducted on the entire human THAP protein family which identifies a second conserved alpha helical region downstream of the THAP domain that is predicted to form coiled coils in all THAP proteins except for THAP10.

It is to be noted that the coiled coil regions predicted in this study have been biochemically and structurally characterized to be important for the homo-dimerization of THAP0 [4, 18], THAP1 [8] and THAP11 [19].

Coiled coil regions are known to mediate oligomerization of proteins [41]. Protein oligomerization is important in many cellular functions like change in cell shape and cell movement by actin [16], endocytosis mediated membrane fission by dynamin dimers [42], signal transduction via membrane receptors by receptor dimerization upon ligand binding, entry and exit from the cell cycle by stable p53 oligomers [14].

Transcription factors are characterized by the presence of DNA binding domains and coiled coil regions [41, 43]. bHLH (basic Helix-Loop-Helix) leucine zippers, one of the most extensively studied family of transcription factors, have a coiled coil region, downstream of a DNA binding domain, which aids in the formation of homotypic dimers. Dimerization of bHLH regulates its functions by enhancing its DNA binding specificity and allowing it to bind two distantly spaced DNA elements [43].

THAP1 [8], THAP5 [44], THAP7 [2] and THAP11 [45] proteins of the human THAP protein family function as transcription regulators. With the identification of the predicted coiled coil region downstream of the conserved DNA binding domain in most THAP proteins and the predicted Nuclear Localization Signal (NLS) [46] in some THAP proteins (Additional file 1: Table S3), we speculate that many other members of the human THAP protein family may be transcription regulators.

## Conclusions

The prediction of evolutionarily conserved coiled coil regions, in all human THAP proteins except THAP10, (upon classification of the THAP protein family into three groups), opens new directions to experimentally explore the cellular functions of THAP proteins. Since coiled coils enable protein oligomerization, this study suggests the possibility of higher order homo- and hetero-oligomer formation by THAP proteins. For example, the presence of a coiled coil region in THAP3 hints at possible interactions with THAP1 [22]. THAP5, a cell cycle inhibitor, may form oligomers via its coiled coil region and act as a decision maker for the cell to continue with the cell cycle or undergo apoptosis similar to p53 [14]. THAP6, which is speculated to function as a transcription factor, may use its predicted coiled coil region to form a leucine zipper. This study may direct further investigations to understand the structure and function of less understood THAP proteins like THAP8 and THAP9. Also, it would be interesting to study the role of THAP10, which is the only THAP family member that does not have a predicted coiled coil region.

## Additional file

Additional file 1: **Figure S1.** Amino acids are represented as (a) *a*, *d* (hydrophobic); *e*, *g* (charged); *b*, *c*, *f* (polar) (b) Hydrophobic (Grey circles), Charged (textured circles), Polar (white circles). **Figure S2.** The color coding in Jalview [47] is described in the legend for Fig. 1. **Figure S3.** (a) The Multiple Sequence alignment was generated using CLUSTAL OMEGA, which represents conserved amino acid residues by an asterisk (*) mark and similarly charged amino acid residues by a colon (:). The most conserved region in all twelve THAP proteins is the amino terminal THAP domain, highlighted in grey. No conservation is found when the predicted alpha helical regions of (b) sTHAP with mTHAP (c) sTHAP with lTHAP (d) mTHAP with lTHAP protein groups are aligned with each other. The Multiple Sequence Alignments for Figures S3b, c and d were generated using CLUSTAL OMEGA and visualized using Jalview. The color coding in Jalview is described in the legend for Fig. 1. **Figure S4.** Protein models generated for (a) Full length THAP protein (b) Corresponding predicted alpha helical region. I TASSER results were viewed using VMD, selecting Ribbon model for secondary structure of proteins with alpha helix (purple), 3_10_ helix (blue), Π- helix (red), beta sheet (yellow), turn (cyan) and coils (white). **Figure S5.** Superposition of THAP7 (green), THAP8 (blue), THAP11 (red). **Figure S6.** The reported crystal structure of THAP11 (yellow) is overlapped (using PyMOL) with the structure of the helical region of THAP11 (cyan) predicted using I TASSER. **Table S1.** Leucine content in THAP proteins and their predicted alpha helical regions. **Table S2.** LOGICOIL and Multicoil predicts higher order oligomer formation. **Table S3.** NLSmapper predicts NLS in THAP0, THAP1, THAP2, THAP4, THAP5, THAP9. The predicted NLS regions in THAP1 and THAP9 overlap with the predicted coiled coil regions of the respective proteins. (DOCX 2200 kb)

## Abbreviations

bHLH: basic Helix Loop Helix
PRKRIR: protein-kinase, IFN-inducible double-stranded RNA dependent inhibitor, and repressor of P58 repressor
THAP: Thanatos Associated Proteins
