# Correction to: Classification of the human THAP protein family identifies an evolutionarily conserved coiled coil region

**DOI:** 10.1186/s12900-019-0105-z

**Published:** 2019-03-29

**Authors:** Hiral M. Sanghavi, Sairam S. Mallajosyula, Sharmistha Majumdar

**Affiliations:** 1 Discipline of Biological Engineering, Indian Institute of Technology Gandhinagar, Gandhinagar, India; 2 Discipline of Chemistry, Indian Institute of Technology Gandhinagar, Gandhinagar, India

**Correction to: BMC Struct Biol (2019) 19: 4**

**https://doi.org/10.1186/s12900-019-0102-2**

In the publication of this article [1], there is an error for one of the contributing authors. This has now been updated in the original article [1].

The error:

Sairam S. Mallajosyala.

It should instead read:

Sairam S. Mallajosyula.
