# Supplementary material for: Classification of the human THAP protein family identifies an evolutionarily conserved coiled coil region
Source: BMC Struct Biol. 2019 Mar 5;19:4. doi: 10.1186/s12900-019-0102-2 (PMC6402169; doi:10.1186/s12900-019-0102-2)
Supplement: Supplementary file 1 — Figure S1. Amino acids are represented as (a) a, d (hydrophobic); e, g (charged); b, c, f (polar) (b) Hydrophobic (Grey circles), Charged (textured circles), Polar (white circles). Figure S2. The color coding in Jalview [47] is described in the legend for Fig. 1. Figure S3. (a) The Multiple Sequence alignment was generated using CLUSTAL OMEGA, which represents conserved amino acid residues by an asterisk (*) mark and similarly charged amino acid residues by a colon (:). The most conserved region in all twelve THAP proteins is the amino terminal THAP domain, highlighted in grey. No conservation is found when the predicted alpha helical regions of (b) sTHAP with mTHAP (c) sTHAP with lTHAP (d) mTHAP with lTHAP protein groups are aligned with each other. The Multiple Sequence Alignments for Figures S3b, c and d were generated using CLUSTAL OMEGA and visualized using Jalview. The color coding in Jalview is described in the legend for Fig. 1. Figure S4. Protein models generated for (a) Full length THAP protein (b) Corresponding predicted alpha helical region. I TASSER results were viewed using VMD, selecting Ribbon model for secondary structure of proteins with alpha helix (purple), 310 helix (blue), Π- helix (red), beta sheet (yellow), turn (cyan) and coils (white). Figure S5. Superposition of THAP7 (green), THAP8 (blue), THAP11 (red). Figure S6. The reported crystal structure of THAP11 (yellow) is overlapped (using PyMOL) with the structure of the helical region of THAP11 (cyan) predicted using I TASSER. Table S1. Leucine content in THAP proteins and their predicted alpha helical regions. Table S2. LOGICOIL and Multicoil predicts higher order oligomer formation. Table S3. NLSmapper predicts NLS in THAP0, THAP1, THAP2, THAP4, THAP5, THAP9. The predicted NLS regions in THAP1 and THAP9 overlap with the predicted coiled coil regions of the respective proteins. (DOCX 2200 kb) [file 12900_2019_102_MOESM1_ESM.docx]

**Additional File**

**Additional Figure S1:** Arrangement of amino acid residues, in an alpha helix that forms coils, in the (a) Heptad pattern and (b) Helical wheel plot


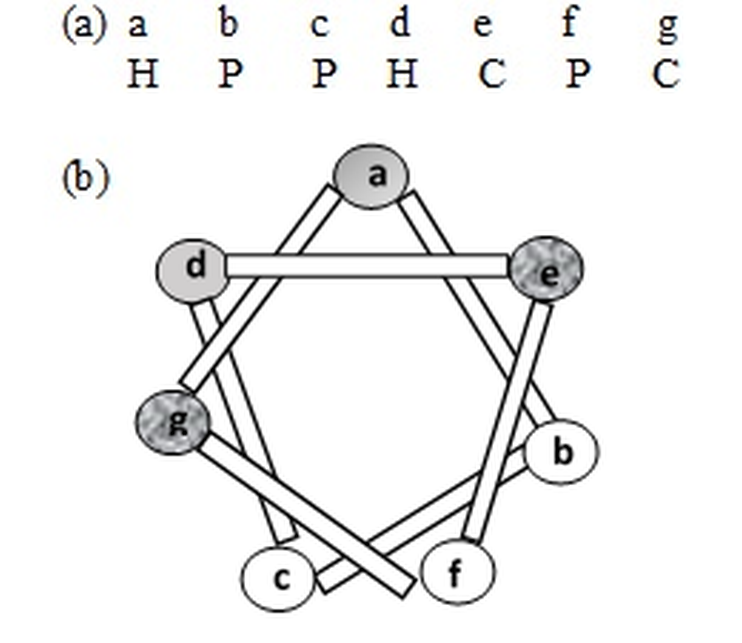


**Additional Figure S2:** Multiple sequence alignment (by Jalview) of the predicted alpha helical region in all human THAP proteins (except THAP10) did not show any conservation of specific amino acid residues

**
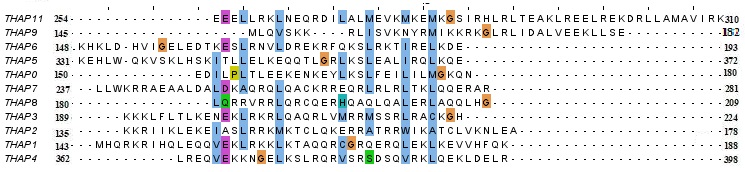
**

**Additional figure 3**

1. Multiple Sequence Alignment of the full length THAP proteins.

| 1. Multiple Sequence Alignment of the predicted alpha helical regions of sTHAP and mTHAP protein groups.   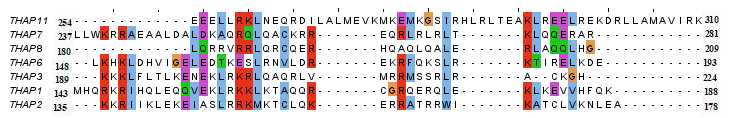     1. Multiple Sequence Alignment of the predicted alpha helical regions of sTHAP and lTHAP protein groups.     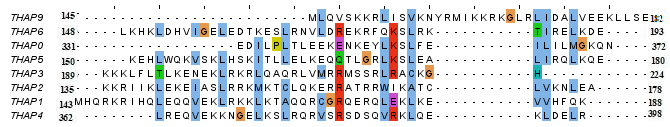   1. Multiple Sequence Alignment of the predicted alpha helical regions of mTHAP and lTHAP protein groups.   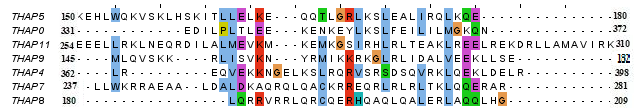  **Additional Figure S4**: Protein structure predictions of THAP proteins generated by ITASSER and visualized  by VMD  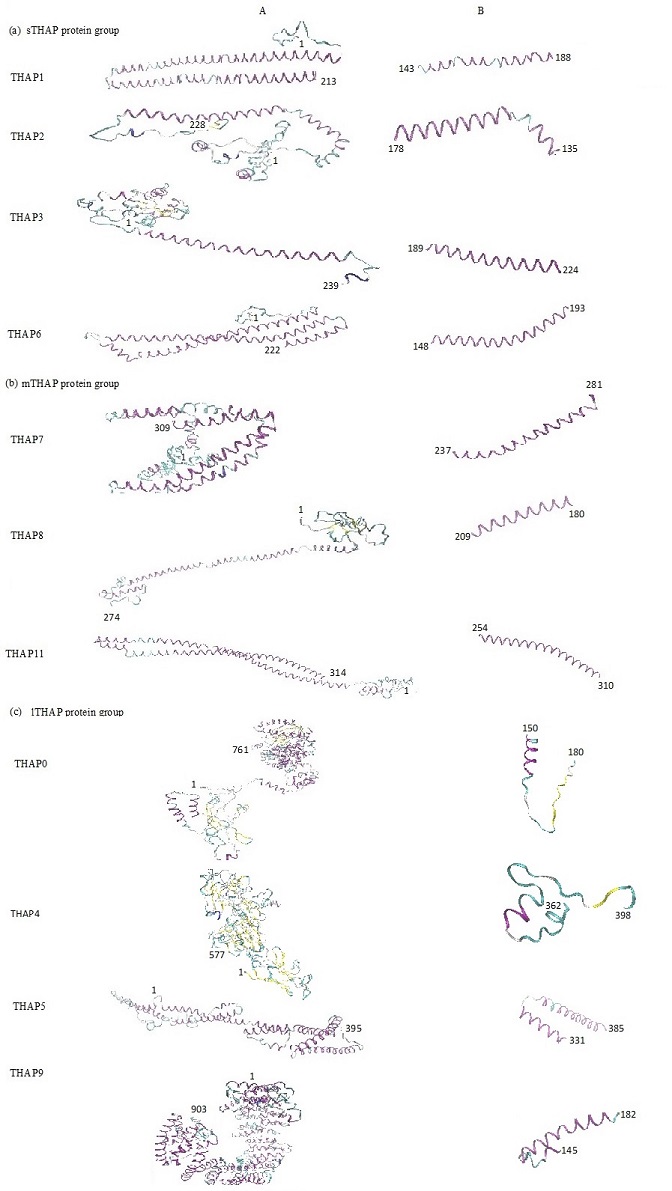 | |
| --- | --- |
|  | |
|  |  |


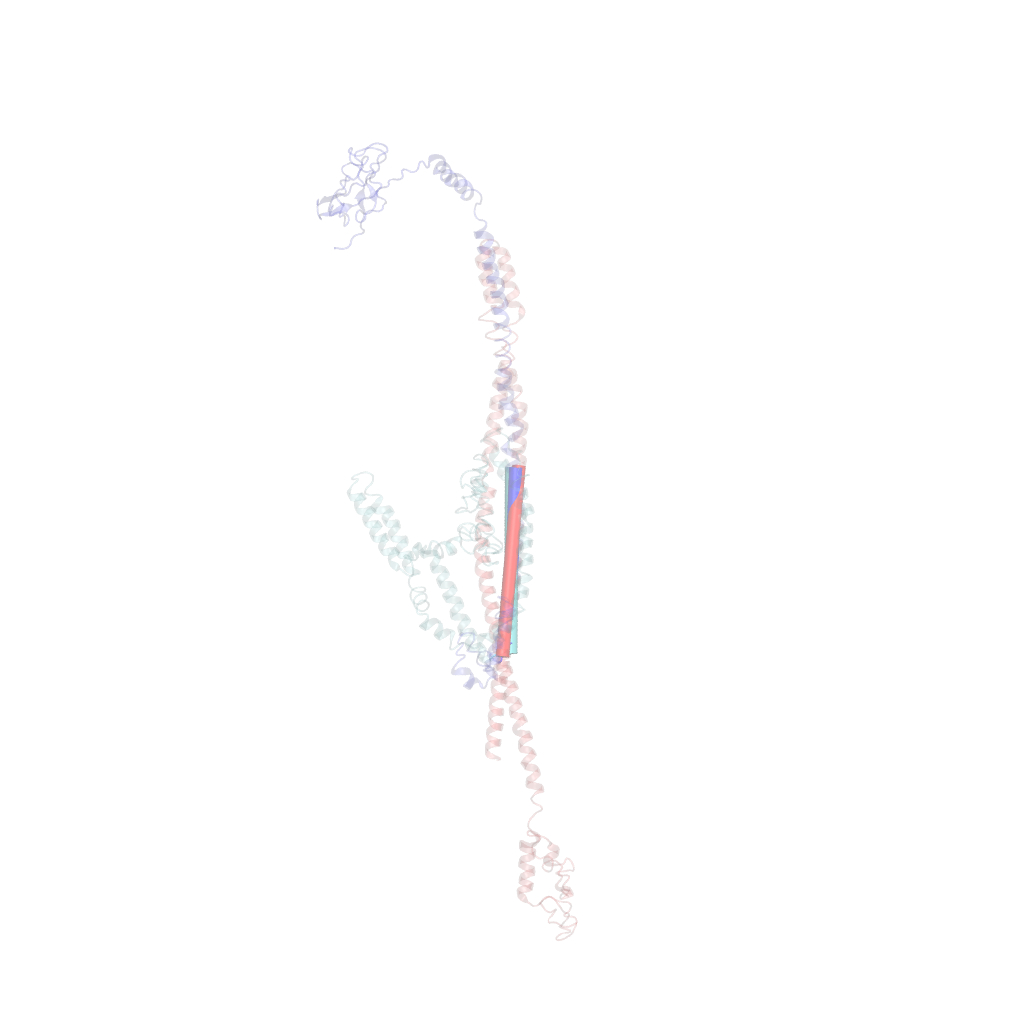


**Additional Figure S5**: Superposition (generated by VMD) of the predicted alpha helical regions(cartoon) of mTHAP proteins, within the rest of each protein (ribbon)

**Additional Figure S6**: Comparison of the reported crystal structure of THAP11 (247-314 amino acid residues) and the predicted alpha helical region in THAP11 (254-310 amino acid residues).


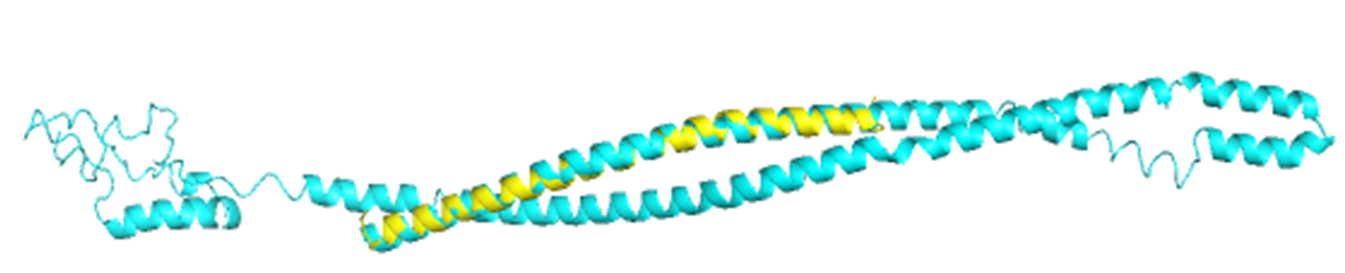


**Additional Table S1:** Leucine content in full length THAP proteins and their predicted alpha helical regions.

| **THAP protein** | **% Leu in full protein** | **% Leu in predicted coiled coil region** | **Most abundant amino acid in the entire protein** |
| --- | --- | --- | --- |
| THAP0 | 11.4 | 21.8 | Glu |
| THAP1 | 8.9 | 10.6 | Lys |
| THAP2 | 11.4 | 11.1 | Lys |
| THAP3 | 9.6 | 17.9 | Arg |
| THAP4 | 6.7 | 15.7 | Ser |
| THAP5 | 9.3 | 19.6 | Glu |
| THAP6 | 9.0 | 14.8 | Lys |
| THAP7 | 9.3 | 19.5 | Arg |
| THAP8 | 10.5 | 18.7 | Pro |
| THAP9 | 12.8 | 17.9 | Ser |
| THAP10 | 5.8 | -- | Ala |
| THAP11 | 8.5 | 18.6 | Gln |

**Average 9.4 16.9**

**Additional Table S2:** Higher oligomeric structure predictions of the predicted alpha helical regions of THAP proteins and their interacting partners (as predicted by STRING database)

| Protein | Oligomeric state of coiled coil as predicted by Logicoil | Probability score | Oligomeric state of coiled coil as predicted by Multicoil | Probability score |
| --- | --- | --- | --- | --- |
| **sTHAP** |  |  |  |  |
| THAP1 | Trimer | 0.9 | Dimer/Trimer | 0.9/0.1 |
| THAP2 | Anti-parallel dimer | 1 | Dimer/Trimer | 0.1/0.1 |
| THAP3 | Parallel dimer | 0.9 | Trimer | 0.01 |
| THAP6 | Tetramer | 1 | Trimer | 0.2 |
|  |  |  |  |  |
| **mTHAP** |  |  |  |  |
| THAP7 | Tetramer | 1 | Dimer/Trimer | 0.4/0.1 |
| THAP8 | -- | -- | Trimer | 0.38 |
| THAP11 | Parallel dimer | 1 | Dimer/Trimer | 0.3/0.1 |
|  |  |  |  |  |
| **lTHAP** |  |  |  |  |
| THAP0 | Trimer | 1 | -- | -- |
| THAP4 | Trimer | 0.9 | Trimer/Dimer | 0.3/0.5 |
| THAP5 | Trimer | 1 | Trimer/dimer | 0.3/0.1 |
| THAP9 | Tetramer | 1 | -- | -- |

| Protein | Oligomeric state as predicted by Logicoil | Probability score | Oligomeric state as predicted by Multicoil | Probability score |
| --- | --- | --- | --- | --- |
| **sTHAP interacting proteins as predicted by STRING database** | | | | |
| PAWR: THAP1 and THAP2 interacting partner | Parallel dimer | 1 | Dimer | 0.9 |
| RRM1: THAP1 and THAP2 interacting partner | Parallel dimer | 0.9 | -- | -- |
| BLZF1: THAP3 interacting partner | Parallel dimer | 1 | Dimer | 0.5 |
| THAP11: THAP2 and THAP3 interacting partner | Parallel dimer | 1 | Dimer | 0.3 |
|  |  |  |  |  |
| **mTHAP interacting proteins as predicted by STRING database** | | | | |
| TAF1B: THAP7 interacting partner | Anti-parallel dimer | 1 | -- | -- |
| PRMT7: THAP8 interacting partner | Anti-parallel dimer | 1 | -- | -- |
| THAP0: THAP11 interacting partner | Trimer | 1 | -- | -- |
|  |  |  |  |  |
| **lTHAP interacting proteins as predicted by STRING database** | | | | |
| FAM171A2: THAP4 interacting partner | Parallel dimer | 0.9 | -- | -- |
| RPL8: THAP0 and THAP5 interacting partner | -- | -- | -- | -- |
| MRPL9: THAP9 interacting partner | Anti-parallel dimer | 1 | Trimer | 0.1 |
| DNAJC3: THAP0 interacting partner | Tetramer 1 | 1 | Trimer | 0.3 |

**Additional Table S3**

Prediction of NLS (by NLSmapper) in some THAP proteins

| **THAP protein** | **Predicted NLS** | **Start residue** | **End residue** |
| --- | --- | --- | --- |
| THAP0 | YENGRKRLKAY | 297 | 307 |
| THAP1 | HQRKRIHQL | 144 | 152 |
| THAP2 | PLDPKRRKEWV | 26 | 36 |
| THAP4 | PIDRKRLKKDVE | 255 | 166 |
| THAP5 | RRGRNNKDRKLSFYPFPLHDKERLEKWLKN | 13 | 42 |
| THAP9 | YRMIKKRKGL | 159 | 168 |

**Raw sequence files**

>THAP0 (NP_004696.2)

MPNFCAAPNCTRKSTQSDLAFFRFPRDPARCQKWVENCRRADLEDKTPDQLNKHYRLCAKHFETSMICRT

SPYRTVLRDNAIPTIFDLTSHLNNPHSRHRKRIKELSEDEIRTLKQKKIDETSEQEQKHKETNNSNAQNP

SEEEGEGQDEDILPLTLEEKENKEYLKSLFEILILMGKQNIPLDGHEADEIPEGLFTPDNFQALLECRIN

SGEEVLRKRFETTAVNTLFCSKTQQRQMLEICESCIREETLREVRDSHFFSIITDDVVDIAGEEHLPVLV

RFVDESHNLREEFIGFLPYEADAEILAVKFHTMITEKWGLNMEYCRGQAYIVSSGFSSKMKVVASRLLEK

YPQAIYTLCSSCALNMWLAKSVPVMGVSVALGTIEEVCSFFHRSPQLLLELDNVISVLFQNSKERGKELK

EICHSQWTGRHDAFEILVELLQALVLCLDGINSDTNIRWNNYIAGRAFVLCSAVSDFDFIVTIVVLKNVL

SFTRAFGKNLQGQTSDVFFAAGSLTAVLHSLNEVMENIEVYHEFWFEEATNLATKLDIQMKLPGKFRRAH

QGNLESQLTSESYYKETLSVPTVEHIIQELKDIFSEQHLKALKCLSLVPSVMGQLKFNTSEEHHADMYRS

DLPNPDTLSAELHCWRIKWKHRGKDIELPSTIYEALHLPDIKFFPNVYALLKVLCILPVMKVENERYENG

RKRLKAYLRNTLTDQRSSNLALLNINFDIKHDLDLMVDTYIKLYTSKSELPTDNSETVENT

>THAP1 (CAG33537.1)

MVQSCSAYGCKNRYDKDKPVSFHKFPLTRPSLCKEWEAAVRRKNFKPTKYSSICSEHFTPDCFKRECNNK

LLKENAVPTIFLCTEPHDKKEDLLEPQEQLPPPPLPPPVSQVDAAIGLLMPPLQTPVNLSVFCDHNYTVE

DTMHQRKRIHQLEQQVEKLRKKLKTAQQRCGRQERQLEKLKEVVHFQKEKDDVSERGYVILPNDYFEIVE

VPA

>THAP2 (AAH08358.1)

MPTNCAAAGCATTYNKHINISFHRFPLDPKRRKEWVRLVRRKNFVPGKHTFLCSKHFEASCFDLTGQTRR

LKMDAVPTIFDFCTHIKSMKLKSRNLLKKNNSCSPAGPSNLKSNISSQQVLLEHSYAFRNPMEAKKRIIK

LEKEIASLRRKMKTCLQKERRATRRWIKATCLVKNLEANSVLPKGTSEHMLPTALSSLPLEDFKILEQDQ

QDKTLLSLNLKQTKSTFI

>THAP3 (AAH92427.1)

MPKSCAARQCCNRYSSRRKQLTFHRFPFSRPELLKEWVLNIGRGNFKPKQHTVICSEHFRPECFSAFGNR

KNLKHNAVPTVFAFQDPTQQVRENTDPASERGNASSSQKEKVLPEAGAGEDSPGRNMDTALEELQLPPNA

EGHVKQVSPRRPQATEAVGRPTGPAGLRRTPNKQPSDHSYALLDLDSLKKKLFLTLKENEKLRKRLQAQR

LVMRRMSSRLRACKGHRGLQARLGPEQQS

>THAP4 (AAH69235.1)

MVICCAAVNCSNRQGKGEKRAVSFHRFPLKDSKRLIQWLKAVQRDNWTPTKYSFLCSEHFTKDSFSKRLEDQHRLLKPTAVPSIFHLTEKKRGAGGHGRTRRKDASKATGGVRGHSSAATGRGAAGWSPSSSGNPMAKPESRRLKQAALQGEATPRAAQEAASQEQAQQALERTPGDGLATMVAGSQGKAEASATDAGDESATSSIEGGVTDKSGISMDDFTPPGSGACKFIGSLHSYSFSSKHTRERPSVPREPIDRKRLKKDVEPSCSGSSLGPDKGLAQSPPSSSLTATPQKPSQSPSAPPADVTPKPATEAVQSEHSDASPMSINEVILSASGACKLIDSLHSYCFSSRQNKSQVCCLREQVEKKNGELKSLRQRVSRSDSQVRKLQEKLDELRRVSVPYPSSLLSPSREPPKMNPVVEPLSWMLGTWLSDPPGAGTYPTLQPFQYLEEVHISHVGQPMLNFSFNSFHPDTRKPMHRECGFIRLKPDTNKVAFVSAQNTGVVEVEEGEVNGQELCIASHSIARISFAKEPHVEQITRKFRLNSEGKLEQTVSMATTTQPMTQHLHVTYKKVTP

>THAP5 (Q7Z6K1.2)

MPRYCAAICCKNRRGRNNKDRKLSFYPFPLHDKERLEKWLKNMKRDSWVPSKYQFLCSDHFTPDSLDIRW

GIRYLKQTAVPTIFSLPEDNQGKDPSKKKSQKKNLEDEKEVCPKAKSEESFVLNETKKNIVNTDVPHQHP

ELLHSSSLVKPPAPKTGSIQNNMLTLNLVKQHTGKPESTLETSVNQDTGRGGFHTCFENLNSTTITLTTS

NSESIHQSLETQEVLEVTTSHLANPNFTSNSMEIKSAQENPFLFSTINQTVEELNTNKESVIAIFVPAEN

SKPSVNSFISAQKETTEMEDTDIEDSLYKDVDYGTEVLQIEHSYCRQDINKEHLWQKVSKLHSKITLLEL

KEQQTLGRLKSLEALIRQLKQENWLSEENVKIIENHFTTYEVTMI

>THAP6 (AAH22989.1)

MVKCCSAIGCASRCLPNSKLKGLTFHVFPTDENIKRKWVLAMKRLDVNAAGIWEPKKGDVLCSRHFKKTD

FDRSAPNIKLKPGVIPSIFDSPYHLQGKREKLHCRKNFTLKTVPATNYNHHLVGASSCIEEFQSQFIFEH

SYSVMDSPKKLKHKLDHVIGELEDTKESLRNVLDREKRFQKSLRKTIRELKDECLISQETANRLDTFCWD

CCQESIEQDYIS

>THAP7 (NP_001008695.1)

MPRHCSAAGCCTRDTRETRNRGISFHRLPKKDNPRRGLWLANCQRLDPSGQGLWDPASEYIYFCSKHFEE

DCFELVGISGYHRLKEGAVPTIFESFSKLRRTTKTKGHSYPPGPAEVSRLRRCRKRCSEGRGPTTPFSPP

PPADVTCFPVEEASAPATLPASPAGRLEPGLSSPFSDLLGPLGAQADEAGCSAQPSPERQPSPLEPRPVS

PSAYMLRLPPPAGAYIQNEHSYQVGSALLWKRRAEAALDALDKAQRQLQACKRREQRLRLRLTKLQQERAREKRAQADARQTLKEHVQDFAMQLSSSMA

>THAP8 (Q8NA92.1)

MPKYCRAPNCSNTAGRLGADNRPVSFYKFPLKDGPRLQAWLQHMGCEHWVPSCHQHLCSEHFTPSCFQWRWGVRYLRPDAVPSIFSRGPPAKSQRRTRSTQKPVSPPPPLQKNTPLPQSPAIPVSGPVRLVVLGPTSGSPKTVATMLLTPLAPAPTPERSQPEVPAQQAQTGLGPVLGALQRRVRRLQRCQERHQAQLQALERLAQQLHGESLLARARRGLQRLTTAQTLGPEESQTFTIICGGPDIAMVLAQDPAPATVDAKPELLDTRIPSA

>THAP9 (NP_078948.3)

MTRSCSAVGCSTRDTVLSRERGLSFHQFPTDTIQRSKWIRAVNRVDPRSKKIWIPGPGAILCSKHFQESD

FESYGIRRKLKKGAVPSVSLYKIPQGVHLKGKARQKILKQPLPDNSQEVATEDHNYSLKTPLTIGAEKLA

EVQQMLQVSKKRLISVKNYRMIKKRKGLRLIDALVEEKLLSEETECLLRAQFSDFKWELYNWRETDEYSA

EMKQFACTLYLCSSKVYDYVRKILKLPHSSILRTWLSKCQPSPGFNSNIFSFLQRRVENGDQLYQYCSLL

IKSMPLKQQLQWDPSSHSLQGFMDFGLGKLDADETPLASETVLLMAVGIFGHWRTPLGYFFVNRASGYLQ

AQLLRLTIGKLSDIGITVLAVTSDATAHSVQMAKALGIHIDGDDMKCTFQHPSSSSQQIAYFFDSCHLLR

LIRNAFQNFQSIQFINGIAHWQHLVELVALEEQELSNMERIPSTLANLKNHVLKVNSATQLFSESVASAL

EYLLSLDLPPFQNCIGTIHFLRLINNLFDIFNSRNCYGKGLKGPLLPETYSKINHVLIEAKTIFVTLSDT

SNNQIIKGKQKLGFLGFLLNAESLKWLYQNYVFPKVMPFPYLLTYKFSHDHLELFLKMLRQVLVTSSSPT

CMAFQKAYYNLETRYKFQDEVFLSKVSIFDISIARRKDLALWTVQRQYGVSVTKTVFHEEGICQDWSHCS

LSEALLDLSDHRRNLICYAGYVANKLSALLTCEDCITALYASDLKASKIGSLLFVKKKNGLHFPSESLCR

VINICERVVRTHSRMAIFELVSKQRELYLQQKILCELSGHINLFVDVNKHLFDGEVCAINHFVKLLKDII

ICFLNIRAKNVAQNPLKHHSERTDMKTLSRKHWSSVQDYKCSSFANTSSKFRHLLSNDGYPFK

>THAP10 (NP_064532)

MPARCVAAHCGNTTKSGKSLFRFPKDRAVRLLWDRFVRGCRADWYGGNDRSVICSDHFAPACFDVSSVIQKNLRFSQRLRLVAGAVPTLHRVPAPAPKRGEEGDQAGRLDTRGELQAARHSEAAPGPVSCTRPRAGKQAAASQITCENELVQTQPHADNPSNTVTSVPTHCEEGPVHKSTQISLKRPRHRSVGIQAKVKAFGKRLCNATTQTEELWSRTSSLFDIYSSDSETDTDWDIKSEQSDLSYMAVQVKEETC

>THAP11 (NP_065190.2)

MPGFTCCVPGCYNNSHRDKALHFYTFPKDAELRRLWLKNVSRAGVSGCFSTFQPTTGHRLCSVHFQGGRK

TYTVRVPTIFPLRGVNERKVARRPAGAAAARRRQQQQQQQQQQQQQQQQQQQQQQQQQQQQQSSPSASTAQTAQLQPNLVSASAAVLLTLQATVDSSQAPGSVQPAPITPTGEDVKPIDLTVQVEFAAAEGAAAAAAASELQAATAGLEAAECPMGPQLVVVGEEGFPDTGSDHSYSLSSGTTEEELLRKLNEQRDILALMEVKMKEMKGSIRHLRLTEAKLREELREKDRLLAMAVIRKKHGM
